# Moonlighting Proteins: Some Hypotheses on the Structural Origin of Their Multifunctionality

**DOI:** 10.3390/ijms262110375

**Published:** 2025-10-24

**Authors:** Juan Cedano, Mario Huerta, Angel Mozo-Villarias, Enrique Querol

**Affiliations:** 1 Departament de Bioquímica i Biologia Molecular, Institut de Biotecnologia i Biomedicina, Universitat Autónoma de Barcelona, Bellaterra, 08193 Barcelona, Spain; 2 Institute of Pathology, University Hospital Heidelberg, 69120 Heidelberg, Germany

**Keywords:** moonlighting proteins, multitasking proteins, moonlighting proteins evolution, non-orthologous gene displacement, non-homologous isofunctional enzymes, fold-switching proteins, protein structure and function, conformational plasticity

## Abstract

Moonlighting proteins—single polypeptides performing multiple, often unrelated functions—are increasingly recognized as key players in human disease and microbial pathogenesis, making their identification crucial for understanding disease mechanisms and developing targeted therapies. This study addresses the unresolved question of how such multifunctionality evolves, focusing on two potential structural mechanisms: Non-Orthologous Gene Displacement/Non-Homologous Isofunctional Enzymes (NOGD/NHIE), where evolutionarily unrelated proteins perform the same function, and Fold-Switching Proteins (FSP), which adopt alternative secondary structures to switch functions without sequence changes. We analyzed the overlap between known human moonlighting proteins (from MultitaskProtDB-II) and curated datasets of NOGD/NHIE (Non-Orthologous Gene Displacement/Non-Homologous Isofunctional Enzymes) and fold-switching proteins (FSPs), using Fisher’s exact test for statistical validation. Moonlighting proteins showed extraordinary enrichment for NOGD/NHIE (19.89% vs. 0.39% in non-moonlighting proteins; odds ratio = 63.1, *p* < 2.2 × 10^−16^) and strong enrichment for FSPs (6.99% vs. 0.26%; odds ratio = 28.7, *p* = 1.13 × 10^−14^), corresponding to ~51-fold and ~27-fold higher risks, respectively. These findings establish intrinsic structural plasticity—whether through evolutionary replacement (NOGD/NHIE) or conformational switching (FSP)—as a central mechanism enabling functional moonlighting in the human proteome. The results suggest that such plasticity facilitates functional innovation while preserving sequence integrity, and that both NOGD/NHIE and FSP features may serve as predictive signatures for identifying novel moonlighting proteins, particularly those with implications for disease mechanisms and therapeutic targeting.

## 1. Introduction

Moonlighting or multitasking proteins refer to those proteins with two or more functions performed by a single polypeptide chain. These alternative functions—historically termed canonical and moonlighting—are often regulated by factors such as cellular localization, cell type, oligomeric state, concentration of ligands, substrates, cofactors, products, or post-translational modifications [1,2,3,4]. While experimental observations—such as aberrant subcellular localization or protein abundance exceeding metabolic needs—can hint at additional roles, moonlighting proteins are typically discovered serendipitously.

The existence of multifunctional proteins complicates the interpretation of genomic and proteomic data, including knock-out/knock-in studies, interactomics, metabolomics, systems biology, and drug development (pharmacokinetics, pharmacodynamics, and toxicity assays). In previous work, we have reported that, according to the results shown in our database MultiTaskDBII (http://thor.uab.cat/MultiTaskDBII, accessed on 15 August 2025) 78% of human moon/lighting proteins are involved in diseases, 48% are targets of current drugs, and 25% of all entries in the database have a moonlighting function related to the virulence of pathogenic microorganisms [5,6,7,8].

A typical moonlighting protein performs a pair of independent, autonomous functions, with the enzyme-transcription factor being the most common combination [5]. From the biochemistry and physiology point of view, both canonical and moonlighting functions could be considered independent of each other. The canonical function refers to the historically first-discovered role, often associated with ancient metabolic processes. However, clinical evidence reveals instances where both functions are closely linked—sometimes synergistically, sometimes antagonistically [9].

### 1.1. Structural and Regulatory Mechanisms of Moonlighting

Regarding orthologous protein counterparts, moonlighting proteins can show from zero to minimal to major changes in sequence, PTMs, etc. In many cases, no amino acid sequence changes are required. For example, 25% of moonlighting proteins in pathogens serve as virulence factors, often simply by being secreted outside the cell [10]. These proteins are frequently conserved in sequence with their human orthologs, enabling immune evasion [7,11]. In other cases, minimal sequence changes suffice—for example, the GroEL of *E. coli* only requires a small change of four amino acids [12].

Post-translational modifications (PTMs) are also key. For example, in GAPDH [13], it can also be a monomer or oligomer with different functions. Many moonlighting proteins contain intrinsically disordered regions (IDRs), and structural changes in these regions can lead to functional shifts [14,15]. In proteins like p53, PTMs such as phosphorylation within IDRs modulate interactions with partner proteins, enabling functional versatility. The existence of moonlighting proteins raises several important questions. (a) How does it acquire a second function? (b) How does it cope with the adaptive conflict instead of giving rise to two different proteins? (c) Why can orthologous proteins present different moonlighting functions in different organisms? (d) Why are some proteins prone to perform multiple moonlighting functions in the same or in different organisms? (i.e., enolase, GA3PD)?

### 1.2. Evolutionary Origins: Key Unanswered Questions

The acquisition of an additional function by a single protein thus offers evolutionary advantages, including limiting the number of genes in the total gene pool, reducing the number of genes needing to be expressed, and, in turn, the number of proteins needing to be synthesized. Yet, a fundamental question remains: how does a protein acquire a second function? In this work, we explore two potential mechanisms: Non-Orthologous Gene Displacement/Non-Homologous Isofunctional Enzymes (NOGD/NHIE) and Fold-Switching Proteins (FSP).

NOGD means that a number of main cell functions can be encoded by non-orthologous genes in different species. At the protein level, the NHIE means that proteins without sequence similarity—and different structural fold—share the function. These phenomena are often detected during genome annotation [16,17]. We hypothesize that a protein recruited to replace a canonical ortholog in another species might retain its original function, thereby becoming a moonlighting protein.

FSPs are proteins that, upon specific biochemical stimuli, remodel stretches of their secondary structures, changing their biological functions [18,19]. We propose that such structural plasticity could enable a single polypeptide to perform multiple functions, thus representing a direct structural origin of moonlighting.

To test these hypotheses, we overlapped our moonlighting protein database with those of NOGD/NHIE and FSP, seeking proteins that match in both function and species.

## 2. Results and Discussion

Despite the limited size of current databases—particularly for FSP and NOGD/NHIE—we identified significant overlaps between moonlighting proteins and those exhibiting NOGD/NHIE or FSP behaviors.

**Figure 1:**
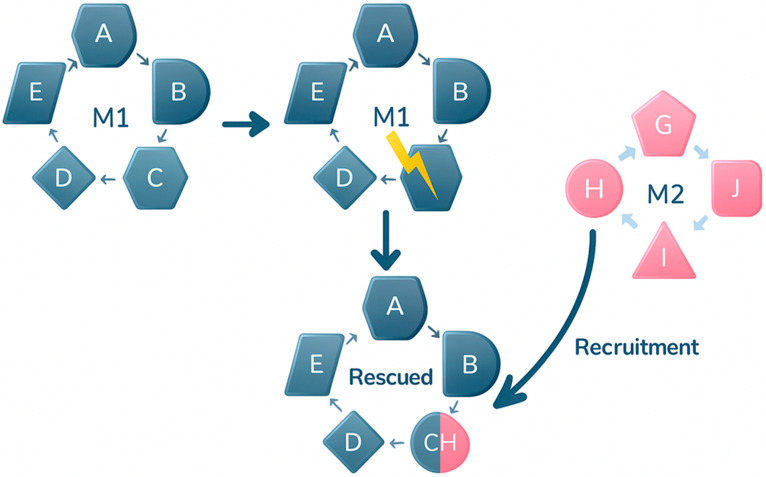
Rescue of a defective metabolic pathway (M1), in blue, through recruitment of an enzyme from an alternative pathway (M2), in pink, that exhibits a moonlighting function. The CH enzyme comes from the M2 metabolic pathway, but now, in its moonlighting function, it allows the M2 pathway to be completed.

**Figure 2:**
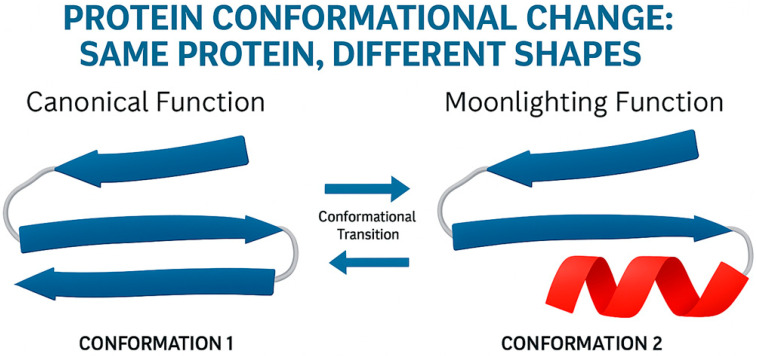
Moonlighting protein function is enabled by conformational change. A single protein can adopt two distinct three-dimensional shapes (Conformation 1 and Conformation 2). Each unique conformation exposes different functional surfaces, allowing the protein to perform its canonical function in one state and a completely separate, moonlighting function in the other. This functional switch is often triggered by specific cellular stimuli.

The loss of an essential enzyme in M1 (red cross) creates a metabolic bottleneck. This failure leads to the recruitment of an enzyme from M2, whose latent moonlighting activity allows it to compensate for the missing enzyme. Thereby, flux through M1 is restored. Figure 1 and Figure 2 show the two mechanisms proposed as the origin of a number of moonlighting proteins.

Table 1 presents 13 matches between our moonlighting protein database and the FSP database. These represent 13 out of 186 FSPs that also appear as moonlighting proteins. The table includes PDB codes, canonical functions and species, moonlighting functions, and structural/functional mapping descriptors relevant to fold-switching regions [18,19].

Table 2 presents 12 human protein matches between our moonlighting protein database and the NOGD/NHIE database. These represent 12 out of 505 NOGD/NHIE proteins that also appear as moonlighting proteins. Of the 505 proteins NOGD/NHIE, we found 52 that match the function and biological species with moonlighting proteins from our database. The complete list of these 52 proteins is in the Supplementary Material. The table includes PDB and UniProt codes, canonical functions and species, moonlighting functions [18,19]. The rest of the examples could be consulted in the Supplementary Material.

Our comprehensive statistical analysis reveals highly significant enrichment of both NOGD (Non-Orthologous Gene Displacement) and FSP (Fold-Switching Protein) characteristics among human moonlighting proteins. For NOGD, Fisher’s exact test demonstrated extraordinary enrichment (odds ratio = 63.1, 95% CI: 42.9–97.8, *p* < 2.2 × 10^−16^). The prevalence of NOGD was 19.89% (37/186) in moonlighting proteins versus 0.39% (94/23,963) in non-moonlighting proteins, corresponding to a 51-fold higher risk (risk ratio = 50.7). A Z-test based on the log-odds ratio yielded Z = 20.996 (*p* = 6.81 × 10^−98^), fully consistent with Fisher’s result.

Similarly, FSP analysis confirmed strong enrichment: 6.99% (13/186) of moonlighting proteins were FSPs compared to 0.26% (39/14,814) of non-moonlighting proteins, representing a 27-fold higher risk (risk ratio = 26.6). Fisher’s exact test gave an odds ratio of 28.67 (95% CI: 15.6–55.2, *p* = 1.13 × 10^−14^), with a corresponding Z-test on the log-odds ratio yielding Z = 10.44 (*p* = 8.76 × 10^−26^).

These results provide robust statistical evidence that both NOGD and FSP mechanisms are strongly associated with moonlighting functionality in the human proteome. Notably, no protein in our dataset exhibited both NOGD and FSP characteristics simultaneously. However, Fisher’s exact test for mutual exclusivity between these two features did not reach statistical significance (*p* = 0.119), suggesting that the observed lack of overlap is not significantly greater than expected by chance given the current sample size and the rarity of both traits.

In summary, our corrected and rigorously validated analysis establishes NOGD and FSP as two distinct, statistically significant mechanisms contributing to protein moonlighting in humans, with fold-switching showing particularly pronounced enrichment in the refined dataset.

### 2.1. Mechanistic Hypotheses for Moonlighting Acquisition

Our results suggest that NOGD/NHIE and FSP mechanisms may contribute to the evolutionary emergence of moonlighting proteins.

In the case of NOGD/NHIE, a protein recruited to perform a function typically carried out by a non-orthologous gene in another species may retain its ancestral function while acquiring a new one. This dual functionality would make it a moonlighting protein. The conservation of the canonical function in orthologs—while a specific lineage acquires an additional role—is consistent with this mechanism. This pattern aligns with a key prediction of the NOGD/NHIE hypothesis: the same protein assumes both roles where others use distinct, non-homologous enzymes.

For FSPs, structural flexibility allows a single polypeptide sequence to adopt alternative folds in response to cellular stimuli, thereby enabling functional switching. Our analysis reveals a 27-fold higher risk (risk ratio = 26.6) of fold-switching among human moonlighting proteins compared to non-moonlighting proteins (6.99% vs. 0.26%), with Fisher’s exact test confirming highly significant enrichment (odds ratio = 28.67, *p* = 1.13 × 10^−14^). This robust statistical support demonstrates that fold-switching is not a marginal phenomenon, but a preferentially exploited molecular mechanism for achieving functional multifunctionality in the human proteome.

Importantly, fold-switching occurs without changes in amino acid sequence or covalent modifications, relying solely on conformational rearrangements of secondary structure elements. This supports the idea that a single polypeptide can perform multiple functions simply by adopting alternative folds, making it a powerful mechanism for functional innovation under selective pressure. Moreover, this association highlights fold-switching as a key mechanism in functional innovation and suggests that FSPs represent a promising route for the identification of novel moonlighting proteins, especially in biomedical contexts.

### 2.2. Strategies for Acquiring Moonlighting Functions: Exploiting Intrinsic Properties

However, there are cases in which a protein can become multifunctional simply by being exported, as in the case of pathogen virulence proteins, or through post-translational modifications, without changing the amino acid sequence.

One paradigmatic example is cytochrome c. In mitochondria, it functions in the electron transport chain. When released into the cytosol due to mitochondrial damage, it triggers apoptosis by forming the apoptosome and activating caspases [20,21]. This illustrates a fundamental mechanism: spatial relocalization as a functional switch. The protein’s presence in a new compartment serves as both a signal and an effector, enabling a new biological role without structural modification.

In this scenario, the simple change in localization of cytochrome c is sufficient to integrate critical information about the cellular state. Its presence in the cytosol not only indicates mitochondrial damage but also directly initiates a programmed cell death response. This strategy is highly efficient from an evolutionary standpoint: it requires no new sensors, additional signaling pathways, or specialized genes. The spatial redistribution of an existing protein serves as both signal and effector.

This mechanism illustrates a central advantage of moonlighting: genetic economy. Instead of encoding specialized proteins to detect each type of cellular stress, the cell can exploit emergent secondary functions of existing proteins, whose localization, concentration, or physical state reveal relevant information. Thus, moonlighting not only reduces the number of necessary genes but also simplifies signaling circuitry, increasing system robustness without adding complexity.

This principle—using a protein’s location or state as functional information—can be considered a primitive yet highly effective form of signal integration, and likely represents one of the earliest origins of protein multifunctionality during evolution.

### 2.3. Redox-Sensitive Functional Switching: Exploiting Catalytic Residues

Another way to exploit an intrinsic property of moonlighting is through the redox sensitivity of specific residues in the active site, particularly cysteines with low pKa that can undergo reversible modifications by reactive oxygen species (ROS). These modifications regulate the primary catalytic activity and can confer a new regulatory or signaling function depending on the cellular redox state, without sequence changes or new protein expression.

A typical example is the peroxiredoxins (Prx). Their canonical role is peroxide reduction via a reactive cysteine. Under oxidative stress, hyperoxidation of this residue induces oligomerization into high-molecular-weight complexes that function as molecular chaperones [22,23,24]. This redox-sensitive conformational switch transforms an antioxidant enzyme into a proteostasis guardian—a self-regulated response to stress.

This functional switch is tightly linked to an intrinsic structural property of Prx: the conformational plasticity induced by the redox state of the catalytic center. Hyperoxidation acts as a redox switch that converts an antioxidant enzyme into a sensor and effector of proteotoxic stress. Thus, the same chemical modification that inactivates its primary function triggers a secondary, essential role in cell survival under stress.

This mechanism represents a highly efficient evolutionary strategy: a single protein integrates information about the cellular redox state and responds directly via a self-induced functional change. No new gene expression or additional signaling cascades are needed—accumulation of ROS alone triggers the switch, allowing Prx to act first as a scavenger and, when overwhelmed, as a proteome protector.

### 2.4. Oligomerization as a Conformational Switch

One of the most frequent structural mechanisms in moonlighting is a change in the oligomeric state. Transitions between monomeric, dimeric, oligomeric, or supramolecular forms can expose or hide functional sites.

For example, GAPDH functions as a glycolytic tetramer but, under stress, dissociates into monomers that translocate to the nucleus and regulate transcription or RNA transport [13,25]. Similarly, Prx switches from decameric peroxidases to filamentous chaperones upon hyperoxidation [24]. These transitions are not merely quaternary changes but involve large-scale conformational reorganizations that redefine functional surfaces [26,27].

This change in oligomerization is a very clear way in which the role of conformational changes in the emergence of moonlighting functions is manifested. However, there may be more subtle forms of conformational change also involved in acquiring these moonlighting functions. Our main contribution is about the FSP.

### 2.5. Fold-Switching Proteins: Subtle Conformational Changes Beyond Oligomerization

FSPs are proteins that, upon specific biochemical stimulus, remodel stretches of their secondary structures, changing their biological functions. This mechanism represents a less evident form of conformational change compared to oligomerization, as it does not necessarily involve changes in quaternary structure or subunit association.

Instead, fold-switching involves the rearrangement of secondary structural elements—such as α-helices converting to β-strands or vice versa—within a single polypeptide chain, leading to distinct tertiary folds and, consequently, different functional surfaces. These structural changes are often triggered by ligand binding, pH shifts, or post-translational modifications, and result in a complete functional switch without gene duplication.

The significant enrichment of FSPs among human moonlighting proteins (27-fold higher risk, odds ratio = 28.67, *p* = 1.13 × 10^−14^) supports the idea that such subtle, yet profound, conformational changes can underlie moonlighting. Unlike oligomerization-based switching, fold-switching does not rely on the exposure of hidden interfaces through subunit dissociation, but on the intrinsic capacity of a protein sequence to adopt multiple stable folds.

This mechanism highlights that moonlighting can arise not only from changes in protein–protein interactions or localization, but from the very plasticity of the polypeptide chain itself. The existence of such proteins challenges the classical “one sequence, one structure, one function” paradigm and supports the view that structural dynamics are central to functional versatility.

### 2.6. Why Is Not Moonlighting More Widespread?

Given its advantages, why is moonlighting not universal? C. Jeffery stated, “Current moonlighting appears to be only the tip of the iceberg” [28]. Limitations include adaptive conflict—optimizing one function may impair another—and experimental detection challenges. A protein is only classified as moonlighting after experimental validation.

Moreover, multifunctionality may depend on the interactomic context. For example, ribosomal protein S10 is moonlighting in E. coli [29], regulating transcription. When aligned with orthologs from nine other microbes, the transcription-regulating motif is more conserved than the rRNA-binding site. Yet, not all orthologs exhibit moonlighting.

This raises a key question: if the moonlighting motif is conserved, why is not the function? The answer may lie in protein–protein interactions. Functional emergence may require a partner protein—such as a regulator or cofactor—that evolves in a specific lineage [30,31]. Thus, moonlighting can be a context-dependent, emergent property of interaction networks [32,33]. The latent functional potential exists, but its realization depends on the interactomic environment.

Moonlighting is not merely an anomaly but a reflection of protein and cellular plasticity. It arises through diverse mechanisms—structural switching, gene displacement, post-translational regulation, and network evolution—underscoring the efficiency and adaptability of biological systems.

## 3. Materials and Methods

Moonlighting proteins were obtained from the database MultitaskProtDB-II [5] (at http://thor.uab.cat/MultiTaskDBII, accessed on 15 August 2025). Fold-Switching Proteins (FSP) were extracted from the supplementary material of Kim and Porter (2021) [19]. NOGD/NHIE proteins were sourced from Omelchenko et al. (2010) [17], specifically Supplementary File 2, Table S2 (available at https://www.ncbi.nlm.nih.gov/Complete_Genomes/AnalEnzymes.html, accessed on 7 June 2025).

Additional data—including UniProt and PDB codes, Pfam domains, and structural mappings—were retrieved from UniProtKB (http://www.uniprot.org, accessed on 15 August 2025) [34] and PDB (http://www.pmc.ncbi.nlm.nih.gov, accessed on 15 August 2025) [35].

In all cases, only those proteins from our DB that exactly match their biological function and biological species with those of the NOGD/NHIE and FSP databases, separately, will be considered true positives.

## 4. Conclusions

Two non-exclusive mechanistic pathways are proposed—though not necessarily the only ones—for the evolutionary emergence of multifunctionality from ancestrally monofunctional proteins.

In the NOGD/NHIE (Non-Orthologous Gene Displacement/Non-Homologous Isofunctional Enzymes) model, a protein with an established structure and primary function is evolutionarily co-opted to perform an additional, lineage-specific role while retaining its original activity. The conservation of the canonical function across orthologs, alongside the appearance of a moonlighting function in specific lineages, strongly supports this as a plausible evolutionary route.

In the fold-switching protein (FSP) model, intrinsic structural plasticity enables a single polypeptide sequence to adopt multiple, stable, and functionally distinct conformations—often in response to cellular cues—thereby facilitating multitasking without gene duplication or sequence divergence.

Statistical analysis reveals a highly significant enrichment of both mechanisms among human moonlighting proteins. NOGD/NHIE: 19.89% vs. 0.39% in non-moonlighting proteins (odds ratio = 63.1, *p* < 2.2 × 10^−16^; ~51-fold higher risk) and FSPs: 6.99% vs. 0.26% (odds ratio = 28.7, *p* = 1.13 × 10^−14^; ~27-fold higher risk).

These results provide robust evidence that conformational switching is a major driver of functional innovation in the human proteome and suggest that FSPs constitute a powerful predictive signature for identifying novel moonlighting proteins—particularly those implicated in disease mechanisms and therapeutic targeting.

As curated databases of moonlighting, NOGD/NHIE, and fold-switching proteins continue to expand, future analyses will further refine and validate these evolutionary and mechanistic models.

## Figures and Tables

**Table 1:** Proteins shared between the moonlighting and Fold Switching Protein (FSP) databases.

FSD PDB Code	Canonical Function and Species	Moonlighting Biological Function	Function Mapping	UniProt Code
5aoeB/5Iy6B	Pneumolysin	Lipid binding & cytolysin	C-ter undecapeptide for membrane binding and cell lysis	Q04IN8
	*S. pneumoniae*		The domains rearrange upon membrane insertion, in particular, alpha-helices in D3 refold to form the transmembrane beta-strands	
			The relative position of domain D4 changes, allowing it to interact with host membranes	
			426–437	
3gmhL/2vfxL	Spindle checkpoint protein Mad2 dimer Human	DNA & RNA binding protein Transcriptional regulation	At 195–205 interaction with CDC20	Q13257
			At 16–191 HORMA domain	
2frhA/1fzpD	SarA Transcription regulator		23 S-diacylglycerol cysteine	P31306
	*Staphyocccus aureus*		23–663 Oligopeptide-binding protein SarA	
4rr2D/319qB	Primase	Bypass oxidative lesions in DNA	Active site at 44 and 109	P49642
	Human		Binding site at 109	
3ifaA/5et5A	Fructose-1,6-biphosphatase	Cytoskeleton and Acrosomal membrane	Active sites at 43, 188, 230	P04075
	Human		Binding sites at 272–274, 301, 304, 364	
4fu4C/4g0Az	Collagenase 3	Gene expression regulation	100–121 Collagenase-like 1	P08253
	Human		397–465 Collagenase-like 2	
			222–236 Collagen binding	
			Fibronectin types at 228–276, 286–334, 344–392	
			Hemopexin at 472–516, 517–563, 565–613, 614–660	
			TIMP2 binding at 414–460	
4dxtA/4dxrA	Sun2 (linker of nucleoskeleton and cytoskeleton)	Signal transduction. Gametogenesis gene-expression	507–717 interaction with SYNE1 and SYNE2	Q9UH99
	Human			
2hdmA/2n54B	Lymphotactin/XCL1	Chemotaxis & inflammatory response	22–114 Lymphotactin	P47992
	(activates the G protein-coupled receptor XCR1)		4–89 Small inducible cytokine A	
	Human		22–114 cathogene G3DSA family	
			32–88 TED domain	
1uxmK/2namA	OxidoreductaseSOD1	RNA-binding protein	2–152 cooper/zing SODC	P00441
	Human		2–151 TED domain	
3ejhA/3m7pA	Fibronectin	Serin protease. Macrophage activation	Glomerulopathy. Spndylometaphyseal dysplasia	P02751
	Human		907–1172 DNA binding	
			52–272 fibrin & heparin binding	
1ceeB/2k42A	CDC42 (Clathrin-mediated endocytosis)	Mitotic spindle orientation	53–88 & 276–311 EF Hands	Q6PJ79
	Human			
2n0aD/2kkwA	Alpha synuclein (tubulin polymerization)	Dopamine regulation	1–116 interaction LIMK1	O94811
	Human		52–207 pfam PF05517 domain	
			49–142 TED domain	
2ougC/2IcIA	RfaH	Translational regulation	3–96 Pfam PF02357 domain	P0AFW0
	*Escherichia coli*			

**Table 2:** Proteins shared between the moonlighting and NOGD/NHIE databases.

Protein Canonical Function and Species	Moonlighting Biological Function(s)	UniProt/PDB Codes
Superoxide dismutase SOD1	RNA-binding protein	P00441/1AZV
Human		
Cyclooxygenase1	Heme-dependent peroxidase	P23219/6Y3C
Human		
Prostaglandin G/H Synthase 1	Cycloxygenase	P23219/6Y3C
Human		
Peroxiredoxin-6	Phospholipase aiPLA2	P30041/5b6m_A
Human		
Serine/threonine protein phosphatase	Dephosphorylating substrates. Chromatin structure. RNA-binding protein	P36873/1IT6
Peroxidase	Phospholipase aiPLA2	P30041/5b6m_A
Human		
Fructose 1,6-biphosphate aldolase	Cytoskeleton and Acrosomal membrane	P04075/1zai_A
Human		
S3 Ribosomal protein	DNA repair	P23396/1WH9
Human	NF-KappB-mediated transcription	
Galactosidase	Cell adhesión and migration	Q6NVH9/1kjl_A
Human		
Glucose-6-phosphate isomerase	Neuroleukin	G6PI_HUMAN/1JLH
Human		
Gluthatione S-transferase	Sperm head proteins involved in zona pellucida binding	Q6FGJ9/Q6FGJDB
Human		
Peptidyl-prolyl isomerase	Cytokine	P62937/1ak4_A
Human		

## Data Availability

The data supporting the reported results are derived from previously published works, including both the authors’ own research and studies by other researchers, all of which are appropriately cited in the bibliography.

## Supplementary Materials

The following supporting information can be downloaded at: https://www.mdpi.com/article/10.3390/ijms262110375/s1.

## Abbreviations

The following abbreviations are used in this manuscript:

NOGD	Non-Orthologous Gene Displacement
NHIE	Non-Homologous Isofunctional Enzymes
FSP	Fold-Switching Proteins
IDR	Intrinsically disordered regions
PTM	Postraductional modifications
Prx	Peroxiredoxins
ROS	Reactive oxygen species
GAPDH	Glyceraldehyde-3-phosphate dehydrogenase
