# Supplementary material for: Moonlighting Proteins: Some Hypotheses on the Structural Origin of Their Multifunctionality
Source: Int J Mol Sci. 2025 Oct 24;26(21):10375. doi: 10.3390/ijms262110375 (PMC12607968; doi:10.3390/ijms262110375)
Supplement: Supplementary file 1 [file ijms-26-10375-s001.zip › ijms-3868321-supplementary.pdf]

Supplementary Material

PROTEINS SHARED BETWEEN THE MOONLIGHTING AND NOGD/NHIE DATABASES

PROTEIN	UniProt & SPS/PDB Codes
Alcohol dehydrogenase	P10127 YEAST/2BI4
Alcohol dehydrogenase	P00334 DROME/1MG5
L-lactate dehydrogenase	P00338 HUMAN/1EZ4
3-alpha hydroxysteroid dehydrogenase (B specific)	P17516 HUMAN/2FVL
Estradiol17-β-dehydrogenase	P14061 HUMAN/1FDS
Estradiol17-β-dehydrogenase	P70694 MOUSE/1Q5M
Testosterone 17- β -dehydrogenase ($NADP^+$)	P37058 HUMAN/2C07
Testosterone 17- β -dehydrogenase ($NADP^+$)	P42330 HUMAN/1S1P
2,4-dienoyl-CoA reductase (NADPH)	Q9NUI1 HUMAN/1W6U
Peroxidase	Q9Y9L0 HUMAN/IMYP
Ferredoxin ($NADP^+$) reductase	Q05783 MYCTU/1LQT
Glutathione transferase	P10620 HUMAN/2H8A
Non-specific protein Tyrosine kinase	P06241 HUMAN/2H8H
Non-specific protein Tyrosine kinase	Q9UIG0 HUMAN/1F62
Phospholipase A2	Q9NZK7 HUMAN/1POE
Phospholipase A2	P47712 HUMAN/1CJY
Lysophospholipase	P47712 HUMAN/1CJY
Aminoacyl tRNA hydrolase	Q980V1 SULSO/1XTY and PTH2 HUMAN/1Q7S
Phosphoprotein phosphatase	P55798 ECOLI/1G5B
Phosphoprotein phosphatase	Q34779 BACSU/2PK0
Phosphoprotein phosphatase	Q9GZU7 HUMAN/ITA0
Phosphoprotein phosphatase	Q8WTR2 HUMAN/1M3G
Alpha N-acetylgalactosamidase	P17050 HUMAN/3H53
Asparaginase	P20933 HUMAN/1APY
Bis(5′-nucleosyl) tetraphosphatase	P50583 HUMAN/1XSA
DUTP diphosphatase	P33316 HUMAN/1Q5H
Adenylate cyclase	Q08828 HUMAN/1CS4

Glucose-6-phosphate isomerase	P06744 HUMAN/1JLH
Chorismate mutase	P19080 BACSU/1COM
DNA toposiomerase	P11387 HUMAN/1LPQ
L-aminoadipate-semialdehyde dehydrogenase	P49419 HUMAN/2J6L
Trans-1,2-dihydrobenzene-1,2-diol dehydrogenase	Q04828 HUMAN/1MRQ
Nitric oxide synthase	P29476 RAT/1TLL
tRNA (guanine-N1-)-methyltransferase	Q32P41 HUMAN/AF-Q32P41
Phosphoserine phosphatase	O82796 ARATH/ AF-O82796-F1
Sphingomyelin phosphodiesterase	P17405 HUMAN/5181
Sphingomyelin phosphodiesterase	Q6UWV6 HUMAN/5TCD
Sphingomyelin phosphodiesterase	O60906 HUMAN/8J2F
Sphingomyelin phosphodiesterase	Q9NXE4 HUMAN/AF Q9NXE4-F1
3',5' cyclic nucleotide phosphodiesterase	Q07343 HUMAN/1XLX
Alpha-L-fucosidase	P04066 HUMAN/1ODU
Steroid delta-isomerase	P14060 HUMAN/AF-P14060-F1
Glutamate dehydrogenase	P39633 BACSU/1GTM
Phospholipase A1	P0A921 ECOLI/1QD5
Poly(ADP-ribose) glycohydrolase	Q9NX46 HUMAN/2FOZ
Poly(ADP-ribose) glycohydrolase	Q86W56 HUMAN/4A0D
Glutaminase (L-Glutamine+ H_2O ⇔LL-Glutamate + NH_3)	Q9UI32 HUMAN/3CZD
Butyryl-CoA dehydrogenase	P26440 HUMAN/1IVH
Nucleoside diphosphate kinase	P56597 HUMAN/8J07
Glycyl-tRNA synthetase	P07814 HUMAN/ AFP07814
Cytidine deaminase	P32320 HUMAN/1MQ0
Piruvate dehydrogenase	P08559 HUMAN/1NI4

Statistical Analysis of Enrichment of NOGD and Fold-Switching Proteins Among Human Moonlighting Proteins

1. Contingency Tables and Descriptive Statistics

1.1. NOGD Proteins vs. Moonlighting Status

	NOGD	No NOGD	Total	%
Moonlighting	37	149	186	19.89%
Non-Moonlighting	94	23,869	23,963	0.39%
Total	131	24,018	24,149	0.54%

- Proportion of NOGD among moonlighting proteins: 18637=0.1989 (19.89%)
- Proportion of NOGD among non-moonlighting proteins: 23,96394=0.00392 (0.39%)
- Risk Ratio (RR): 0.003920.1989=50.7
  → Moonlighting proteins are ~51 times more likely to be NOGD than non-moonlighting proteins.

1.2. Fold-Switching Proteins (FSPs) vs. Moonlighting Status

	FSP	No FSP	Total	%
Moonlighting	13	173	186	6.99%
Non-Moonlighting	39	14,775	14,814	0.26%
Total	52	14,948	15,000	0.35%

- Proportion of FSP among moonlighting proteins: 18613=0.0699 (6.99%)
- Proportion of FSP among non-moonlighting proteins: 14,81439=0.00263 (0.26%)
- Risk Ratio (RR): 0.002630.0699=26.6
  → Moonlighting proteins are ~27 times more likely to be fold-switching than non-moonlighting proteins.

2. Inferential Statistics

All statistical tests were performed using R (v4.3.0). Two-sided tests were used throughout.

2.1. NOGD Enrichment Analysis

- Fisher's Exact Test:
  - Odds Ratio (OR) = 63.1
  - 95% Confidence Interval: [42.9 – 97.8]
  - p-value $< 2.2 \times 10^{-16}$
- Z-test for log(Odds Ratio) (Woolf method):
  - Z = 20.996
  - p-value $= 6.81 \times 10^{-98}$

Both tests confirm extremely significant enrichment of NOGD proteins among moonlighting proteins.

2.2. Fold-Switching Protein (FSP) Enrichment Analysis

- Fisher's Exact Test:
  - Odds Ratio (OR) = 28.67
  - 95% Confidence Interval: [15.6 – 55.2]
  - p-value $= 1.13 \times 10^{-14}$
- Z-test for log(Odds Ratio):
  - Z = 10.44
  - p-value $= 8.76 \times 10^{-26}$

3. Interpretation

- Moonlighting proteins are strongly enriched for both NOGD (OR = 63.1, $p < 2.2e^{-16}$) and fold-switching (OR = 28.7, $p = 1.1e^{-14}$) characteristics.
- The risk ratios (~51-fold for NOGD; ~27-fold for FSPs) indicate a substantive biological effect, not merely a statistical artifact.
- The concordance between Fisher's exact test and asymptotic Z-tests supports the robustness of these findings.

4. Key Takeaway for Main Text

Human moonlighting proteins are significantly enriched in both NOGD (OR = 63.1, $p < 2.2 \times 10^{-16}$) and fold-switching proteins (OR = 28.7, $p = 1.1 \times 10^{-14}$), highlighting structural disorder and conformational plasticity as potential central mechanisms underlying functional moonlighting in humans.
